# Belief-consistent information is most shared despite being the least surprising

**DOI:** 10.1038/s41598-024-56086-2

**Published:** 2024-03-13

**Authors:** Jacob T. Goebel, Mark W. Susmann, Srinivasan Parthasarathy, Hesham El Gamal, R. Kelly Garrett, Duane T. Wegener

**Affiliations:** 1 Department of Psychology, Ohio State University, Columbus, OH USA; 2 Present Address: Department of Psychology, Vanderbilt University, Nashville, TN USA; 3 Department of Computer Science and Engineering, Ohio State University, Columbus, OH USA; 4 Faculty of Engineering, University of Sydney, Sydney, NSW Australia; 5 School of Communication, Ohio State University, Columbus, OH USA

**Keywords:** Novelty, Surprise, Belief consistency, Sharing, Human behaviour, Computer science

## Abstract

In the classical information theoretic framework, information “value” is proportional to how novel/surprising the information is. Recent work building on such notions claimed that false news spreads faster than truth online because false news is more novel and therefore surprising. However, another determinant of surprise, semantic meaning (e.g., information’s consistency or inconsistency with prior beliefs), should also influence value and sharing. Examining sharing behavior on Twitter, we observed separate relations of novelty and belief consistency with sharing. Though surprise could not be assessed in those studies, belief consistency should relate to less surprise, suggesting the relevance of semantic meaning beyond novelty. In two controlled experiments, belief-consistent (vs. belief-inconsistent) information was shared more despite consistent information being the least surprising. Manipulated novelty did not predict sharing or surprise. Thus, classical information theoretic predictions regarding perceived value and sharing would benefit from considering semantic meaning in contexts where people hold pre-existing beliefs.

## Introduction

People encounter countless pieces of information throughout their daily lives, and online interpersonal communication plays a major role in this exposure. This presents a critical challenge; given limited time and cognitive resources, what information is deemed ‘valuable’ enough to share? Information theory suggests that information derives its value from how novel or surprising it is perceived to be^1,2^. Furthermore, the prevailing information theoretic approach is semantic-free, meaning that it considers only whether the information is known or not; the theory does not take into account how the information semantically fits with existing beliefs. Implicit in this perspective is the assumption that novelty and surprise are consistently positively correlated.

We argue, however, that the attributes of novelty and surprise depend on the subjective meaning of the information, specifically its consistency with prior beliefs. We refer to this as a context-sensitive perspective. Novelty is best defined as the degree to which information is not yet stored in memory, whereas surprise is an emotional response to information that contrasts with one’s expectation formed through prior experience^3,4^. From this perspective, novel information is not necessarily surprising. For example, people are rarely surprised by new evidence that their existing beliefs are correct. Because novelty and surprise can operate independently, they can, under some conditions, exhibit opposing relations with sharing behavior. Distinguishing novelty and surprise as antecedents of information diffusion can bolster efforts to predict large-scale social movements^5–7^, implement outreach campaigns and PSAs^8^, and combat the prevalence of online misinformation^9,10^, among other applications^3^.

There is ample evidence that information novelty can promote sharing. Antismoking messages containing novel arguments have been found to exhibit greater retransmission^11^, and novel health and political information regarding COVID-19 spread faster than familiar information^12^. Scholars have even speculated that the reason false information posted on Twitter tends to be shared more widely than true information is that those falsehoods tended to be more novel and more likely to elicit surprise^13^. Other research has linked surprise with information sharing^14^. News articles that evoke surprise go viral more often^15^, and potentially surprising information is often shared with limited concern for its accuracy^16^.

Although novelty and surprise are often positively correlated, they are distinct concepts such that surprise can be derived from sources other than novelty^3,17^. For instance, stories with expectation-violating twists are more surprising^18^, and persuasion research demonstrates that people are more likely to find information surprising when it contradicts rather than validates their pre-existing attitudes^19,20^. Classical information theory obscures this difference by asserting that surprise evoked by information (termed “surprisal”) is inversely proportional to its a priori probability of occurrence^3,21^. When dealing with human experience, this probability would be the probability of previously encountering the information. However, Bayesian theory is more nuanced. It defines surprise as a response to the *discrepancy* between prior beliefs and beliefs implied by the information^22–24^. Thus, a piece of information might be objectively “novel” (i.e., never seen previously) but elicit little surprise due to its consonance with prior beliefs. Similarly, information that is inconsistent with one’s beliefs might be perceived as surprising even if it is not novel^3^. In line with this idea, a significant body of research treats belief consistency as separable from perceived novelty^25–27^.

Despite their distinct antecedents, novelty and surprise have rarely been examined independently in relation to information sharing, potentially due to the prevailing notion from semantic-free information theory that connects novelty and surprise. In some cases, surprise has been used as a proxy for measuring novelty^13,28,29^. However, surprise, stemming from inconsistency of information with beliefs might sometimes influence information value in ways that contrast the predicted effects of novelty. Whereas novelty has been associated with greater sharing^13^, information running counter to strong beliefs (i.e., surprising information) is often viewed with skepticism^30^ and shared less than information consistent with one’s beliefs or attitudes^31–37^. Yet, it seems likely that belief-inconsistent information would generally be viewed as more surprising. Classic information theory has largely ignored such possibilities^1,2^. Past research has not directly tested whether the effects of objective novelty and belief consistency on surprise (and sharing) are separable^37^.

Factors other than novelty can clearly guide information sharing. For instance, despite observing that novel information was more likely than non-novel information to spread via email, some research found that non-novel content was shared more than novel content via social media^29^. This again suggests that factors other than novelty, perhaps including belief consistency, might exert greater influence on sharing intentions, at least in some contexts. Most Americans say that accuracy should be a very important part of their sharing decisions^37^. Yet the illusory-truth effect tells us that familiarity—the opposite of novelty—promotes the perception that information is accurate^36,38,39^ and can promote sharing of fake news^14,40,41^.

**Figure 1:**
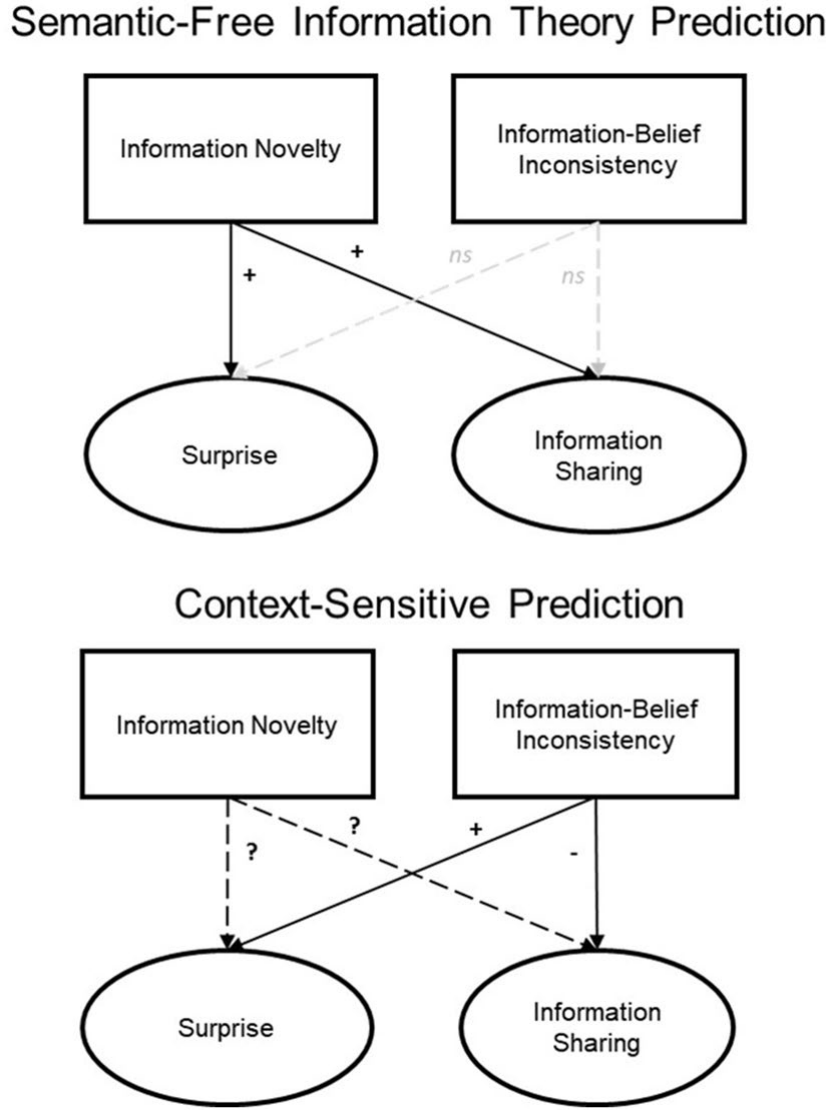
Comparison between semantic-free and context-sensitive perspectives on the relations connecting information novelty and information-belief inconsistency to perceptions of surprise and information sharing.

In sum, we pit predictions based on the semantic-free perspective against those derived from the context-sensitive perspective (see Fig. 1). We argue that the effects of novelty and surprise on information sharing are context-sensitive and need not always work in parallel. Surprise is not solely based on information novelty; instead, we argue that it is also influenced by semantic consistency with prior beliefs. The first and most important aim of the present research is thus to confirm that belief consistency is a reliable predictor of information sharing and that it has an effect that is independent of information novelty. To this end, we observed information sharing behavior on Twitter, assessing the extent to which tweets’ novelty and belief consistency, two potential antecedents of surprise, drive sharing. In addition, to assess the causal mechanisms behind these relations, we conducted two experiments testing (a) whether information’s match (or mismatch) with one’s prior beliefs induces surprise (independent of information novelty) and (b) whether belief consistency and novelty might differentially impact information sharing and perceived value.

## Results

### Twitter analyses

We conducted several analyses to assess the independent influence of information novelty and information-belief consistency on information sharing via Twitter.

#### Novelty associated with sharing

To assess whether novel tweets are shared more often than less novel tweets, we first identified tweets that should differ in novelty but not belief consistency. Operationalizing novelty with Twitter data is challenging; Twitter does not report which tweets a user has seen. Given this constraint, we opted to operationalize novelty by capitalizing on the fact that the first information shared about a new event will necessarily be more novel than information about that same event shared later. Although there is no guarantee that users will have seen any one tweet, earlier tweets are, in aggregate, more novel than subsequent tweets about the same event. Extending this idea, we operationalized novelty using the time since the initial tweet about a discrete event—tweets occurring soon after the event should be quite novel, whereas tweets occurring longer after the event should be less novel.

We collected politically relevant tweets from politically neutral media sources that contained clearly pro-liberal or pro-conservative information about discrete events. To test whether novel tweets (that is, those posted closer to the time of the event) were shared more frequently than less novel tweets, we constructed a general linear mixed-effects model predicting the number of retweets each tweet received using the number of minutes following the first tweet about the event to which a given tweet pertained. We also included the source of a tweet and the event to which it pertained as random factors to account for variability across sources and events. Finally, because the number of retweets is a count variable whose distribution is likely to be Poisson in form, we used Poisson modeling to estimate the effects of tweet timing on the number of retweets.

Results were consistent with novelty being associated with information sharing. The more time that had passed since the first tweet about an event was posted, the fewer retweets the information received, *b* = − 3.44, *se* = 0.17, *Z* = − 20.602, *p* < 0.001, 95% Confidence Interval (CI) [− 3.776, − 3.121].

#### Ideological consistency associated with sharing

**Figure 2:**
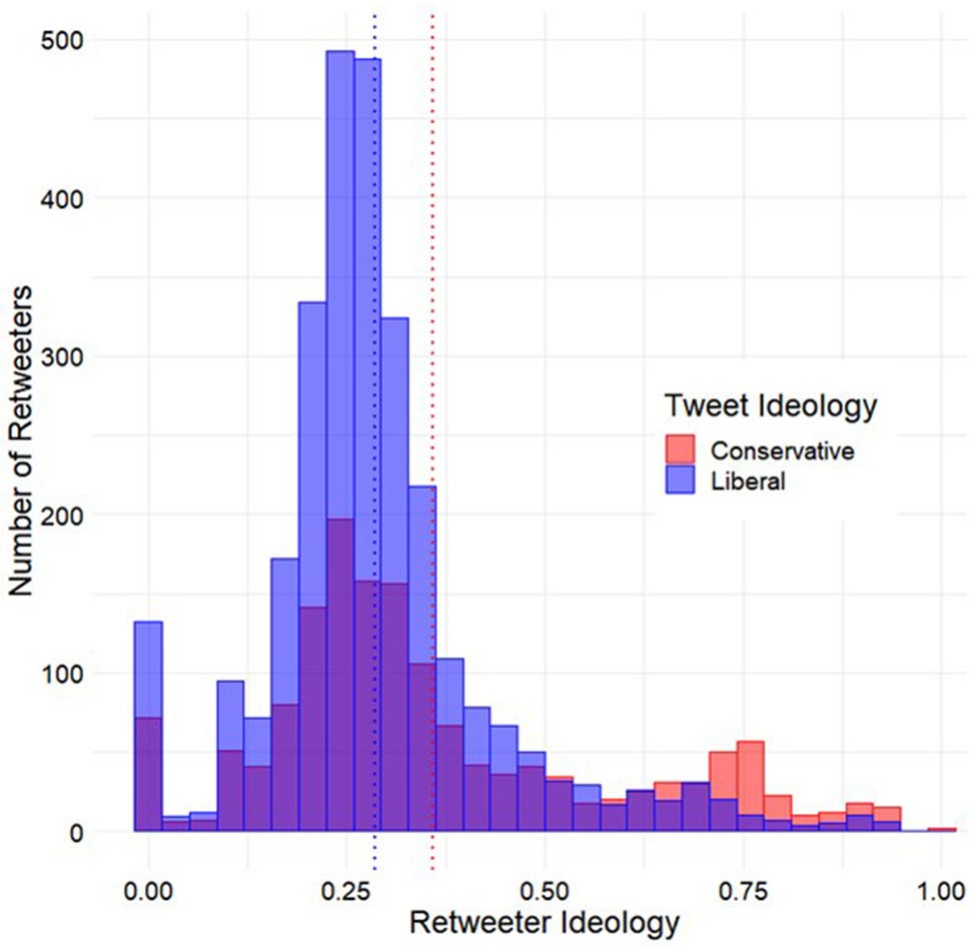
Distributions of retweeter ideology estimates as a function of tweet ideology, sampling tweets from the novelty analysis. The blue dotted line represents the mean ideology of retweeters of liberal-aligned tweets, whereas the red dotted line represents that mean ideology for retweeters of conservative-aligned tweets.

To examine whether tweets containing belief-consistent information are more likely to be shared than those containing belief-inconsistent information, we examined whether individuals were more likely to share tweets expressing views that aligned with their political ideology. We manually coded the slant of each tweet, and we estimated users’ ideology as the average ideology of the U.S. politicians they followed on Twitter (see Methods for more detail)^42^. To facilitate the intensive analysis required for each retweeter, a subsample of 43 Tweets was drawn from the original set of 223 Tweets used in the novelty analysis. Because the ideology estimation method relied on observing which U.S. politicians a user followed, only users following at least one politician were included in these analyses. This resulted in a final sample of 4413 retweeters. Consistent with expectations, an independent samples t-test confirmed that users who retweeted liberal-aligned tweets were, on average, significantly more liberal (*M* = 0.29, *SD* = 0.15) than users who retweeted conservative-aligned tweets (*M* = 0.36, *SD* = 0.22; see Fig. 2), *t*(4411) = − 13.10, *p* < 0.001, Mean Difference 95% CI [− 0.083, − 0.062].

**Figure 3:**
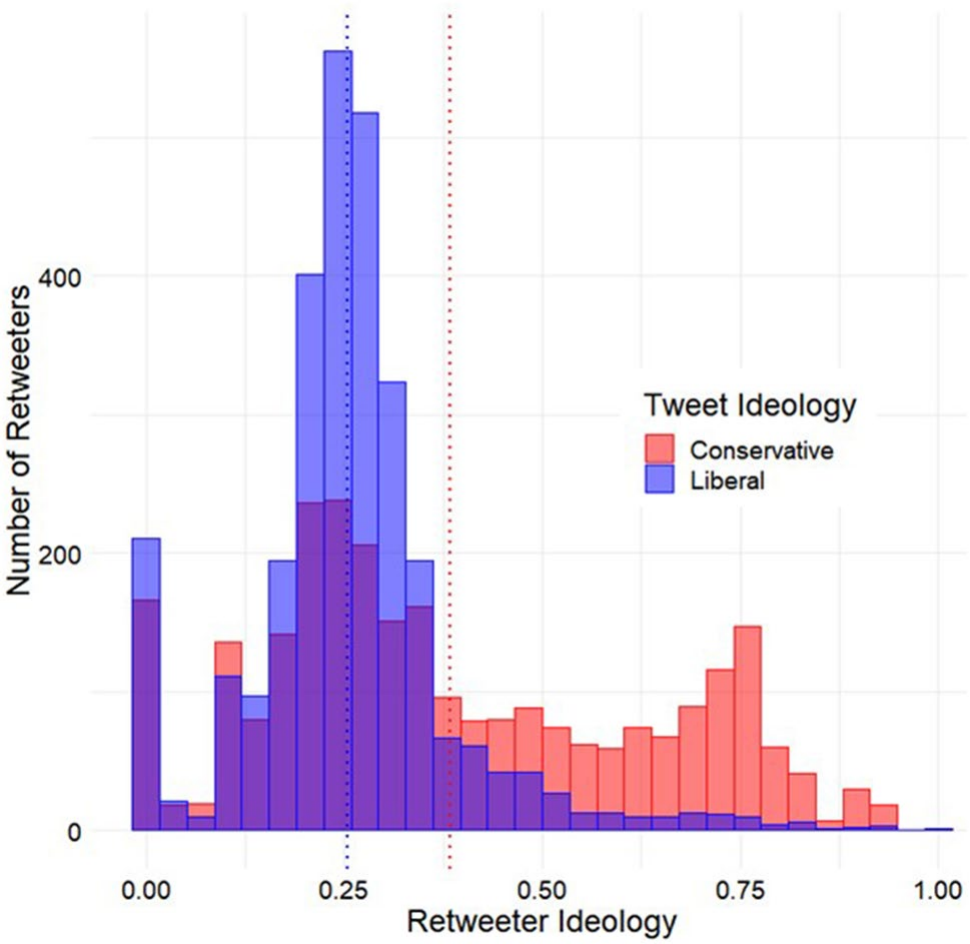
Distributions of retweeter ideology estimates as a function of tweet ideology from a second, independent sample of tweets. The blue dotted line represents the mean ideology for retweeters of liberal-aligned tweets, whereas the red dotted line represents that mean ideology for retweeters of conservative-aligned tweets.

To ensure the robustness of these results, we replicated these analyses using a second dataset by collecting an additional, non-overlapping sample of 64 political tweets that provided clearly pro-liberal or pro-conservative content (again from politically neutral media sources and about discreet events). This dataset included 5724 retweeters. Results were consistent with what we observed in the first analysis. Users who retweeted liberal-aligned tweets were significantly more liberal (*M* = 0.26, *SD* = 0.13) than users who retweeted conservative-aligned tweets (*M* = 0.38, *SD* = 0.24; see Fig. 3), *t*(5722) = − 25.42, *p* < 0.001, Mean Difference 95% CI [− 0.138, − 0.119].

Taken together, these analyses support the notion that for a given tweet, both its novelty and its ideological consistency—which is related to belief consistency^43^—are plausible predictors of information sharing. Most importantly, more widespread sharing of tweets aligned rather than misaligned with one’s beliefs might suggest that information value does not solely derive from novelty/surprise of the information. Rather, consistency of the information with existing beliefs might enhance the perceived value of the information despite the likelihood that such information would not be perceived as particularly surprising.

These at-scale analyses have several limitations that make it difficult to draw strong conclusions about the roles of novelty and belief consistency in information sharing. First, because Twitter does not make available information about which tweets a given individual has seen (unless they interact with it in some way), it is impossible to determine novelty at the individual level. Relatedly, like previous research using Twitter data, we do not have access to Twitter's internal algorithm for surfacing tweets to users. Thus, we do not know to what extent the patterns observed here are the product of individuals’ reactions to the novelty of a tweet versus the way the Twitter algorithm incorporates novelty. Although the freshness of information seems likely to correspond with its novelty for those who encounter it, our proxy for novelty is far from ideal. Thus, it is not possible with these data to directly compare the influences of novelty and belief consistency on sharing.

Second, ideological consistency is an imperfect proxy for beliefs. Liberals and conservatives often hold different beliefs thanks to ideological perceptual biases^43^, but ideology is a noisy proxy for belief. For example, conservatives might be less likely than liberals to believe that human activity is contributing to climate change, but a majority of conservatives do believe that human activity contributes^44^. Additionally, and quite importantly for our purposes, there are no good measures of surprise using Twitter data. Thus, we cannot tell whether belief-consistent information was, in fact, less surprising than belief-inconsistent information or whether (lack of) surprise would relate to information sharing. Finally, sharing decisions about political events are likely driven by more than just relevant prior beliefs, including self-presentational concerns^45,46^, defensiveness^47^, and social identity or the desire to bond with others^46^. Examining sharing decisions in a more controlled situation where one has no ulterior motives would therefore be desirable. Much prior research explores how broad individual ideological alignment, partisanship, or core values impact decisions to transmit information^48^, including most work on selective sharing^32–34,36,37^.

Thus, we conducted a pair of experiments that manipulated initial beliefs in order to provide more direct and causal information about belief consistency predicting information sharing. A goal of the experiments was to more cleanly instantiate participants’ initial beliefs to better isolate the effect of belief consistency per se on sharing intentions. The two experiments also allowed us to directly manipulate information novelty in addition to belief consistency, as well as measure subjective novelty and surprise.

### Experiments

We conducted a pair of experiments designed to address the limitations of the observational Twitter study. In both experiments, we induced a set of beliefs. We manipulated both the novelty and the belief consistency of a message that we presented to participants, and we asked participants to assess the message and to decide whether to share it. The experiments used different topics as a form of stimulus sampling, and Experiment 2 included additional outcome measures.

We adapted a paradigm from prior research examining the perseverance of newly established beliefs^49,50^. We induced initial beliefs by presenting participants with information about an unfamiliar topic. In Experiment 1, the information presented (fictional) evidence that risk takers make either better or worse firefighters. In Experiment 2, the information presented (fictional) evidence that a country should or should not be allowed to join the European Union (EU). We employed topics about which people did not already have strong pre-existing beliefs in order to facilitate our manipulations of belief consistency and novelty. This enables us to more clearly examine the causal impact of beliefs unconfounded with prior experience and knowledge.

Next, we asked participants to play the part of an editorial assistant at a news organization by reviewing two new pieces of information. Each piece of information either supported or contradicted their initial beliefs (the belief consistency manipulation) and either had or had not been seen before (the novelty manipulation). Participants rated how novel and surprising both pieces of information were and indicated whether the information should be transmitted to the newspaper editor. The second experiment also included a measure of perceived information value.

#### Experiment analysis strategy

Most analyses used multiple regression, with exceptions noted below. Save for the manipulation check, predictors in these analyses were always the same: outcomes were predicted by the consistency condition (i.e., whether the information provided in the update supported or contradicted the conclusion established in the earlier phase), as well as the novelty condition (i.e., whether information contained in the update had been previously encountered or not).

#### Manipulation check

Our manipulations were effective in instilling the desired initial beliefs. In Experiment 1, those in the riskiness-is-good-for-firefighting condition indicated that they thought riskiness was significantly better for firefighting than those in the riskiness-is-bad-for-firefighting condition, *b* = 22.62, *se* = 1.55, *t*(224) = 14.58, *p* < 0.001, 95% CI [19.566, 25.682], *r* = 0.70. In Experiment 2, those provided with favorable country attributes indicated that they thought the country should be allowed to join the EU significantly more than those provided with less favorable country attributes, *b* = 18.60, *se* = 1.21, *t*(299) = 15.34, *p* < 0.001, 95% CI [16.211, 20.982], *r* = 0.66.

#### Belief inconsistency associated with surprise

Belief-consistent information was rated as less surprising than belief-inconsistent information in both Experiment 1, *b* = − 0.61, *se* = 0.08, *t*(223) =  − 7.39, *p* < 0.001, 95% CI [− 0.766, − 0.444], *r* = 0.44, and Experiment 2, *b* = − 0.70, *se* = 0.10, *t*(298) =  − 7.22, *p* < 0.001, 95% CI [− 0.893, − 0.511], *r* = 0.39. In contrast, the novelty manipulation did not impact surprise in Experiment 1, *b* = − 0.08, *se* = 0.08, *t*(223) =  − 1.04, *p* = 0.30, 95% CI [− 0.246, 0.076], *r* = 0.07, or Experiment 2, *b* = 0.02, *se* = 0.10, *t*(298) = 0.25, *p* = 0.80, 95% CI [− 0.166, 0.215], *r* = 0.01. Of note, participants’ ratings of novelty and their ratings of surprise were not positively correlated in either study as would be expected from semantic-free information theory. To the contrary, a small negative correlation was observed in both Experiment 1, *r*(224) = − 0.24, *p* < 0.001, and Experiment 2, *r*(299) = − 0.18, *p* = 0.002. An alternative analysis strategy to examine the impact of belief consistency would be to inspect interactions between information and belief directions. See Supplementary Data 1 for such analyses on each of the outcomes.

#### Belief-consistent information more valued and shared

**Figure 4:**
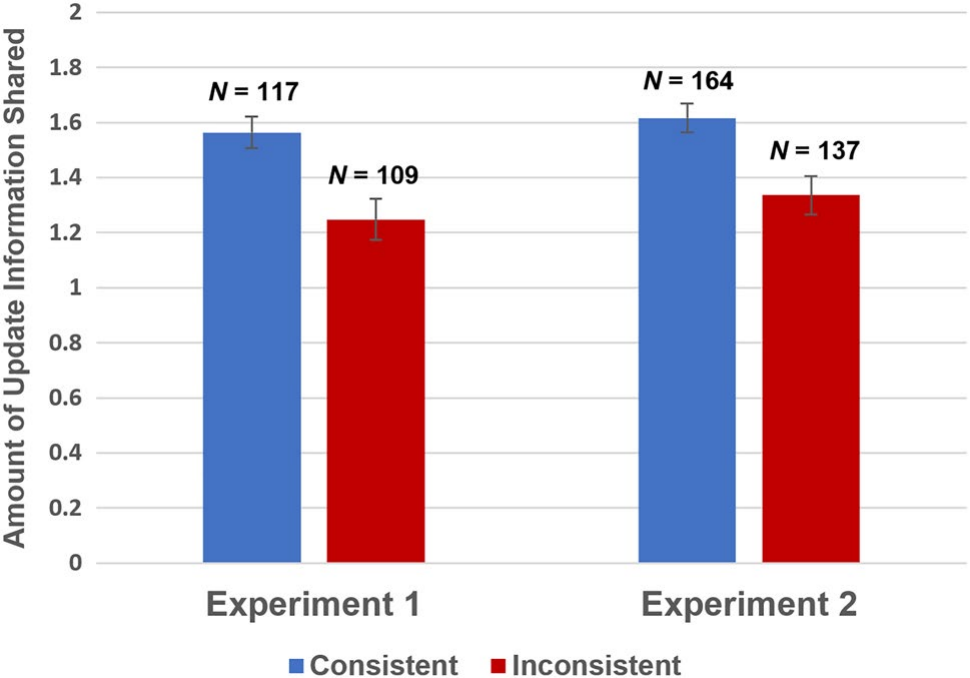
Effect of update information consistency on the amount of update information shared. Each bar indicates the mean amount of update information shared by participants. Error bars represent standard error.

In Experiment 2, belief-consistent information was rated as more valuable than belief-inconsistent information, *b* = 0.20, *se* = 0.07, *t*(298) = 2.77, *p* = 0.006, 95% CI [0.058, 0.341], *r* = 0.16. In contrast, the novelty manipulation had no significant effect on perceived value, *b* = 0.03, *se* = 0.07, *t*(298) = 0.43, *p* = 0.67, 95% CI [− 0.110, 0.172], *r* = 0.02. This preference for belief-consistent information parallels the pattern of actual sharing decisions (see Fig. 4).

Because our information sharing outcome variable is a count of the amount of update information that was shared, these analyses were conducted using ordinal logistic regression. Recall that participants were exposed to two pieces of information within the update, such that they could decide to transmit zero, one, or two pieces of update information to the editor. Participants were more likely to share information that was belief consistent rather than belief inconsistent in both Experiment 1, *b* = 0.40, *se* = 0.13, *t*(222) = 3.07, 95% CI [0.147, 0.662],* Odds Ratio *(*OR*) = 1.50, and Experiment 2, *b* = 0.37, *se* = 0.12, *t*(297) = 3.05, 95% CI [0.132, 0.602], *OR* = 1.44. The effect of the novelty manipulation on the amount of update information shared did not reach significance in Experiment 1, *b* = − 0.01, *se* = 0.13, *t*(222) = − 0.10, 95% CI [− 0.269, 0.243], *OR* = 0.99, or in Experiment 2, *b* = − 0.09, *se* = 0.12, *t*(297) = − 0.76, 95% CI [− 0.327, 0.144], *OR* = 0.91.

#### Novelty manipulations undetected

To explore why our novelty manipulation failed to impact sharing intentions, we tested whether *subjective* novelty ratings for the update information differed across conditions. The novelty manipulation had no significant effect on perceived novelty in Experiment 1, *b* = 0.08, *se* = 0.10, *t*(223) = 0.73, *p* = 0.47, 95% CI [− 0.129, 0.280], *r* = 0.05, or in Experiment 2, *b* = − 0.03, *se* = 0.08, *t*(298) = − 0.43, *p* = 0.67, 95% CI [− 0.190, 0.122], *r* = 0.02, suggesting that participants did not consistently identify information they had not seen before as being more novel than information they had seen before. Moreover, the belief consistency manipulation had no significant impact on perceived novelty in Experiment 1, *b* = 0.13, *se* = 0.10, *t*(223) = 1.28, *p* = 0.20, 95% CI [− 0.072, 0.337], *r* = 0.09, or in Experiment 2, *b* = 0.15, *se* = 0.08, *t*(298) = 1.91, *p* = 0.06, 95% CI [− 0.005, 0.309], *r* = 0.11. Thus, it seems that influences of belief consistency are unique to surprise rather than uniformly influencing both surprise and perceived novelty.

## Discussion

**Figure 5:**
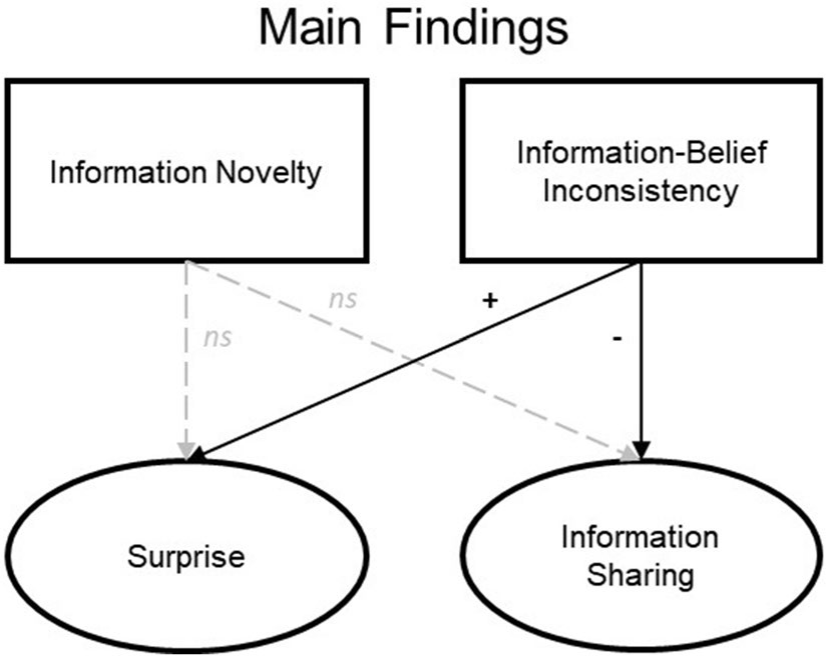
Conceptual depiction of the main findings from Experiments 1 and 2.

In the field and in the lab, we found that information’s consistency with prior beliefs reliably predicts information sharing, in line with the context-sensitive perspective (and contrary to the semantic-free perspective). Our experimental findings also suggest that novelty and surprise are distinct concepts with unique consequences for information sharing (see Fig. 5). Unexpectedly, although our Twitter analyses were consistent with information novelty predicting sharing, our experiments that provide more control and a more precise manipulation of novelty offered no evidence that novelty was related to sharing.

Our experimental findings diverged in notable ways from what semantic-free information theory would predict^1,2^. First, novel (previously unencountered) information was not shared more than information that was not novel (previously encountered). Furthermore, *less* surprising information was most likely to be transmitted, directionally opposite to assumptions in information theory. The only positive predictor of sharing was information-belief consistency, and such a preference for belief-consistent information might be even stronger when information pertains to more closely-held beliefs (e.g., political views^37,48^). These results align with other work demonstrating the relevance of contextual factors to information spread within networks^51^.

Also of interest was participants’ inability to distinguish between information that was objectively novel versus not novel in their ratings of information novelty or surprise. Our novelty manipulation was unequivocal; information was either previously encountered or not. This insensitivity to objective novelty evident in participants’ subjective appraisals might explain why the novelty manipulation failed to impact sharing or perceived value. On the other hand, participants’ levels of surprise in response to belief-consistent versus belief-inconsistent information showed that participants did react differently to information based on its belief consistency. Information inconsistent with prior beliefs was perhaps more surprising because it was deemed less likely to be true^52^. As such, it seems that people might react more to information’s belief consistency and base their sharing decisions more on that factor than on the information’s novelty. Notably, our belief consistency manipulation had no impact on perceived novelty, following prior research distinguishing these constructs^25–27^.

The large-scale observational data have a major limitation: Twitter’s recommendation algorithm is a black box. We know that Twitter promotes content based on recency and engagement^53^, and it is at least possible that it also prioritizes novelty. The details of the algorithm, however, are not publicly available. This means that we have no way of assessing the extent to which the association between novelty and sharing in our Twitter data is due to individual preferences versus Twitter’s algorithms. This is an important limitation, but it does not undermine our claim that sharing predictions based on semantic-free information theory are incomplete. First, we note that the extant literature provides ample evidence that novelty promotes sharing^11–16,47^. Given this, it is likely that at least some of the effects of novelty observed here are, in fact, attributable to individual preferences. Second, and more importantly, we demonstrate that belief-inconsistency (which is correlated with surprise) is a significant negative predictor of sharing regardless of the level of information novelty—regardless of whether it is a by-product of individual preference or algorithmic recommendation.

The experiments have limitations, too. First, they both utilized unfamiliar topics about which participants should have had relatively weak (if any) pre-existing beliefs. This was desirable in that it allowed us to better control the variables of interest (i.e., the novelty of new information and its consistency with beliefs established in the prior phase), ensuring that factors outside of our experimental procedures would not interfere with the results. However, the extent to which the present experimental findings generalize to contexts where people do hold strong prior beliefs, such as the political (as was examined in our Twitter analyses) or health^54^ domains, remains unclear. Future research should examine these experimental results using topics where pre-existing beliefs exist to assess the present findings’ generalizability to those contexts. Similarly, because the information sharing tasks in the present experiments were hypothetical, there were no real consequences to the participants or others associated with what information was or was not shared. Real world information sharing is often consequential, especially if the shared information is incorrect or leads to incorrect conclusions. Though our tasks were designed to prompt participants to consider such consequences, they were nevertheless hypothetical. As such, future research should continue to examine information sharing in settings where decisions can have real consequences, similar to our analyses of belief consistency in Twitter retweets. The experimental scenario also likely minimized participants’ self-presentational and affiliative motives, both core drivers of information sharing^46,47,55,56^. Research should attempt to replicate our findings with these concerns made salient.

Taken as a whole, this work demonstrates that when modeling information sharing, we must distinguish between novelty and surprise. Using Twitter data, we show that the people sharing tweets are more likely to agree than disagree with the political leanings of the tweets regardless of the information’s novelty. The pair of experiments further distinguish between novelty in surprise by showing that a novelty manipulation was not related to surprise and that (lack of) surprise was associated with sharing (of belief-consistent information), whereas novelty was not associated with information sharing. Surprise, in the form of belief consistency, is the best predictor of information sharing, but in a direction opposite to that assumed by semantic-free information theory (i.e., less surprise was associated with more sharing).

More generally, these results suggest that context-sensitive predictions are more accurate than those based on semantic-free information theoretic principles. Indeed, recent research documents that false partisan news propagates more quickly in ideologically segregated networks, seemingly because the implausibility of ideologically-inconsistent false news discourages diffusion in mixed networks^57^. This accords with other work on the propagation of misinformation in polarized “echo chambers” online^58–61^. One important ingredient for constructing such models thus appears to be the judicious combination of information theoretic concepts with a principled approach for studies of subjective perceptions that allow for capturing more relevant variables in the context of interest.

The present research opens a number of doors for future investigation. First, research should examine whether other factors separate from, but correlated with, information novelty predict information sharing. Namely, our Twitter analysis is consistent with the idea that information that is ‘fresh’ (e.g., released close in time to a focal events’ occurrence) is more likely to be shared than less fresh information. If novelty in the form of “freshness” reliably predicts sharing, it would be interesting to consider what aspects of information freshness might lead to greater sharing. One possibility is that more recent events, whether or not they are particularly novel to perceivers, are more likely to be current topics of conversation than older events. If so, people might be motivated to engage with fresher tweets to stay included in current conversations^62^. People also might be more likely to see fresher tweets that attract more engagement than less fresh tweets, amplifying the reach of those fresher tweets. Testing such possibilities in an experimental context would be beneficial.

These theoretical insights also have practical implications. For example, the findings involving belief consistency could suggest that framing information as consistent with the prior beliefs of target populations might increase its likelihood of retransmission. For instance, broadcasted messages (e.g., PSAs) might benefit from framing arguments as consistent with personally important moral foundations^63^, values^64^, or political ideologies^65^ of target audiences. Likewise, the current findings suggest that it might not be safe to assume that novel information will be spread by recipients. This might be especially important in contexts where information is rapidly changing, such as in crisis situations^66^, where the novelty of new guidance might not be sufficient to ensure its spread. In such cases, additional steps, such as framing the guidance to be as consistent with prior-held beliefs as possible, might be beneficial. Further research should explore these possibilities. Our findings are also consistent with how pre-existing beliefs hinder corrections of political misinformation^67,68^. Whether corrections are also less effective at attenuating the *transmission* of belief-consistent misinformation is a ripe area for contemporary research^36,40,69–72^.

Complementing observational and experimental work, agent-based models^73^ offer a lens through which to evaluate factors that might bolster interventions to curtail the spread of (mis)information at scale^74–78^. That is, such models allow for clear manipulations of individual-level factors (e.g., prior beliefs and information exposure) to explore macro-level outcomes (e.g., large-scale information diffusion). Social media field experiments are another complementary direction for future work that can lay the foundation for real-world interventions^79,80^. These approaches could help discern whether corrections or inoculations targeting people for whom circulating misinformation is likely to be belief consistent are ideal. For example, such interventions might only be worthwhile to the extent that messages emphasize consistency with prior beliefs while debunking the specific piece of misinformation.

Overall, the present findings suggest that the meaning of information can modulate the typical effects of novelty and surprise expected from a straightforward application of semantic-free information theory. Specifically, we found that information-belief consistency (a key determinant of surprise) and novelty have distinct influences on sharing intentions. Belief consistency is associated with low levels of surprise but high levels of sharing. As such, our conclusion is that care must be exercised in applying information theoretic principles when sharing occurs in contexts where people hold pre-existing beliefs relevant to information they receive. From a big picture perspective, it seems important to consider the semantics of information when approaching real-world information sharing problems.

## Methods

### Twitter analyses methods

#### Data

Using the Twitter API, we downloaded 223 tweets and the associated metadata, including when each message was posted and the number of times it was retweeted (*M* = 339.68, *SD* = 1022.00). Each tweet pertained to a distinct, discrete event that occurred at a specific point in time. The tweets were posted by relatively non-partisan news organizations such as Reuters, the Associated Press, Newsweek, and Axios. These organizations were selected using ratings from AllSides, an independent organization that specializes in assessing media bias across the political landscape^81^. All news outlets included in the present study were deemed politically neutral at the time of data collection. This was done to make it unlikely that the followers of the respective accounts would be systematically biased in the liberal or conservative directions. Third, we sought events about which non-partisan news sources tweeted multiple times.

All 223 tweets were used for the novelty analyses. For the belief consistency analyses, we also downloaded user IDs of the thousands of users who retweeted the selected messages and all those whom they followed. That is, for each of roughly 100 tweets (43 in the first analysis and 64 in the second), each retweeter (~ 300 per tweet) followed on average approximately 2000 accounts (that were used to determine the ideology of each retweeter). Thus, the final datasets for the belief consistency analyses included over sixty million data points.

#### Sharing

For analyses addressing novelty and sharing, we operationalized information sharing as the number of retweets a given tweet received. When examining relations between belief consistency and sharing, however, we classified the ideological leanings of people who retweeted clearly pro-liberal versus pro-conservative tweets.

#### Novelty

We used time, in minutes, between when the tweet being evaluated was posted and when the first tweet about the same event was posted as a proxy measure for information novelty. This time variable was standardized for use in the analysis.

#### Ideological consistency

To assess the ideological consistency of the tweets, we compared the ideological slant of the post to the ideological orientation of the user sharing it. If the two ideologies matched, we labeled the tweet ideologically consistent. To assess the ideological slant of the tweets, two members of the research team coded by consensus whether each tweet was liberal- or conservative-aligned. That is, we examined whether the message espoused or promoted liberal or conservative positions (see Supplementary Data 2 for examples; the full list of Tweet IDs is available on OSF^82^). Ideological consistency was based on the political slant of the tweet, not the event.

To assess retweeters’ ideological orientation, we adapted a method from prior research that builds on the assumption that people tend to follow politicians who share their political ideology^42^. For example, those who follow the most liberal/conservative members of the U.S. Congress tend to hold more ideologically extreme views. First, we randomly selected eight events (4 liberal-aligned and 4 conservative-aligned) from the sample of 223 tweets used in the novelty analysis and extracted all the tweets pertaining to that event (43 tweets total). We also performed an identical analysis on a second, separate sample of 64 tweets that met the same criteria as the first.

Next, we obtained estimates of each U.S. Senate and House member’s ideology from govtrack.us, a non-partisan organization that tracks congressional events and member voting activity^83^. These estimates are derived from a member’s voting behavior and a comparison between that behavior and the behavior of other members of congress. Ideology estimates range from 0 (most liberal) to 1 (most conservative). We also collected both the professional and personal Twitter user IDs of all US Senate and House members.

Finally, we determined whether each retweeter followed one or more U.S. House and/or Senate members. If so, we assigned that retweeter with an ideology score that was the simple average of the ideology scores of the politicians whom they were following. Those users who did not follow a member of Congress were excluded from the analyses. This resulted in 4413 and 5724 retweeters being included in the first and second analyses, respectively.

### Experiment methods

#### Participants and design

Two hundred ninety-four Introduction to Psychology students enrolled in Ohio State University’s Research Experience Program participated online in Experiment 1 in exchange for partial course credit. Forty-eight participants were excluded from analysis due to being duplicate responses or failing to complete all of the key measures, resulting in an analyzed sample of 226 (Age: *M* = 18.90, *SD* = 1.60; Gender: 38.9% male, 59.3% female, 1.8% other or no response). Three hundred four workers on Amazon’s Mechanical Turk were recruited to participate in Experiment 2 in exchange for $1.50. Of these, 3 participants were excluded from analyses using the same criteria as Experiment 1, resulting in an analyzed sample of 301 (Age *M* = 39.45, *SD* = 11.71; Gender: 52.3% male, 46.7% female, 1% non-binary).

Both studies employed a 2(Update Information Novelty: Not Novel vs. Novel) X 2(Update Information Consistency with Initial Beliefs: Consistent vs. Inconsistent) X 2(Belief Direction: Riskiness is Good for Firefighting/Country Should be Allowed to Join the EU vs. Riskiness is Bad for Firefighting/Country Should Not be Allowed to Join the EU) between-subjects design.

#### Procedure

After providing informed consent, participants in Experiment 1 were told they would take on the role of an editor’s assistant at a news organization. Their goal was to decide to transmit as much information to the editor as necessary to understand the conclusion drawn from the total evidence uncovered by reporters. They read an initial interview transcript under the premise that they would soon receive an update from the reporter. The interview was reportedly conducted with a fire chief and concerned the merits of risky behavior for effective firefighting. With this transcript, participants’ beliefs about the relation between riskiness and firefighting success were manipulated. Participants were randomly assigned to see a transcript in which the fire chief, as well as most embedded supporting information (5 of the total 7 pieces), suggested that riskiness was either beneficial or detrimental. The other 2 pieces of information were in opposition to the primary position taken by the chief. Our experimental paradigm drew from prior studies involving newly established beliefs^49,50^.

Participants then reported their belief about this relation on a continuous scale before viewing the reporter’s “update.” Within the update, another source presented two pieces of evidence either supporting or opposing the conclusion from the prior interview. As such, the consistency of the update information with participants’ initial beliefs was manipulated between subjects. Additionally, this supporting or opposing information was either the same as information participants had seen in the transcript (familiar information) or different from the information in the transcript (novel information).

After again reporting their belief, participants rated each piece of information included within the update on the degree to which it was novel and surprising. They were then asked to report which information they deemed necessary to transmit to the newspaper editor.

Study 2 employed a largely identical procedure, save a few key distinctions. Chiefly, the reporter’s topic was modified to concern whether or not an unnamed country should be allowed to join the European Union (adapted from prior research^84^). Participants also completed a four-item measure of “value” for each piece of update information before making their transmission decisions. Lastly, the ratings of novelty and surprise occurred following, rather than prior to, the sharing phase to ensure that our findings were not due to increased salience of these features at the time of sharing.

#### Dependent variables

*Novelty Ratings*. In both studies, participants’ assessments of how novel they found each piece of information in the update were assessed using three items. For pieces of information explaining the link between riskiness and firefighting success, they were asked to indicate the extent to which they had encountered that explanation before (*1—I’ve Never Encountered This Explanation Before* to 7—*I’ve Definitely Encountered this Explanation Before*), how familiar they were with that explanation (*1—Not at All Familiar* to 7—*Extremely Familiar*), and how often they had seen the explanation before (*1—I Have Never Heard or Seen This Explanation* to 7—*I Have Heard or Seen This Explanation Very Often*). Responses to each of these three items were averaged together for each piece of information, and then these two indexes were averaged together to create a single index of perceived novelty of the update information. The composite scales demonstrated adequate reliability in both Experiment 1 (*α* = 0.90, *M* = 4.29, *SD* = 1.56) and Experiment 2 (*α* = 0.90, *M* = 5.07, *SD* = 1.38).

*Surprise Ratings.* In both studies, participants indicated how surprising they found the update information to be on three items. Participants were asked to what extent they found each of the two explanations to be surprising (*1—Not at All Surprising* to 7—*Very Surprising*) and unexpected (*1—Not at All Unexpected* to 7—*Very Unexpected*), and whether each explanation caught them off guard (*1—Not at All* to 7—*Very Much*). Responses to the three items were averaged together for each of the pieces of information and the two indexes were averaged together to create a single index of surprise. The composite scales demonstrated adequate reliability in both Experiment 1 (*α* = 0.90, *M* = 2.94, *SD* = 1.37) and Experiment 2 (*α* = 0.96, *M* = 3.37, *SD* = 1.82).

*Belief ratings and belief rating shifts*. In Experiment 1, participants’ beliefs about whether riskiness is good or bad for firefighting were assessed using one item immediately following the transcript. This item asked participants to provide their estimate of the direction and strength of the relation between success as a firefighter and risk taking. Participants were reminded that a positive relation means that high riskiness is associated with success as a firefighter, and a negative relation means that high riskiness is associated with failure as a firefighter. Responses were given on a single slider scale (*− 50—Highly Negative Relationship* to *50—Highly Positive Relationship*). Beliefs were assessed again using the same scale after participants read the update information. To assess shifts in beliefs from before exposure to the update information to after, a difference score was calculated by subtracting the post-update information belief ratings from the initial belief ratings. As such, positive values of the difference score indicated that beliefs shifted in the direction of believing that riskiness is good for firefighting, whereas negative values corresponded to shifts in beliefs in the direction of believing that riskiness is bad for firefighting (*M* = − 0.06, *SD* = 35.33).

In Experiment 2, similar items were used except they assessed beliefs about whether the country should or should not be allowed to join the EU (− *50—The Country Should NOT be Allowed to Join the EU* to *50—The Country SHOULD be Allowed to Join the EU*). A difference score was computed in the same fashion. Positive values indicated shifts in beliefs in the direction of believing that the country should be allowed to join, whereas negative values indicated shifts in the direction of believing that the country should not be allowed to join (*M* = − 3.12, *SD* = 23.74).

*Perceived value of the update information*. In Experiment 2, four items assessed how valuable participants found each of the two pieces of information within the update to be. Participants reported the extent to which they thought the information was worth sharing (*1—Not at All Worth Sharing* to 7—*Extremely Worth Sharing*), valuable (*1—Not at All Valuable* to 7—*Very Valuable*), and important to their overall conclusion, (*1—Not at All Important* to 7—*Very Important*). Participants also rated whether they would include the information in their report to their editor (*1—Definitely No* to 7—*Definitely Yes*). All responses to the four items for each piece of information were averaged together and then the resulting two indexes were averaged to create a single overall index of perceived value. The composite scale demonstrated adequate reliability (*α* = 0.94, *M* = 5.62, *SD* = 1.26).

*Intention to share information*. In both studies, participants were presented with a list of all the information they had encountered from both the initial transcript and the update. Participants were asked to indicate which pieces of information they would like to include with their report to their editor by dragging each piece of information from the list into a box labeled “include” or a box labeled “exclude.” We assessed participants' desire to share the update information by counting how many of the pieces of information from the update they placed in the include box. Participants could include both explanations from the update, just one explanation, or neither of them in Experiment 1 (*M* = 1.41, *SD* = 0.71) and Experiment 2 (*M* = 1.49, *SD* = 0.76).

### Ethics

Human subjects research was determined exempt from full review and approved by The Ohio State University Human Research Protection Program (HRPP) under protocol number 2021E1208 (Federalwide Assurance (FWA) for the Protection of Human Subjects Number: 00006378). All research was performed in accordance with relevant guidelines and regulations. Informed consent was obtained from all human research participants.

### Supplementary Information

Supplementary Information.

## Data Availability

Twitter and experimental data files have been made available in the Open Science Framework [https://osf.io/wy3hp/?view_only=b4f98d8ddfd746a1a71df412ee829a6a]^83^. In accordance with content redistribution policies for Twitter Content, we are restricted to sharing only Tweet IDs and User IDs.

## Code availability

R code used in analyses has been made available in the Open Science Framework [https://osf.io/wy3hp/?view_only=b4f98d8ddfd746a1a71df412ee829a6a]^83^.
