# Supplementary material for: Belief-consistent information is most shared despite being the least surprising
Source: Sci Rep. 2024 Mar 13;14:6109. doi: 10.1038/s41598-024-56086-2 (PMC10937659; doi:10.1038/s41598-024-56086-2)
Supplement: Supplementary file 1 — Supplementary Information. [file 41598_2024_56086_MOESM1_ESM.pdf]

Belief-Consistent Information is Most Shared Despite Being the Least Surprising

Jacob T. Goebel,[1*] Mark W. Susmann,[2] Srinivasan Parthasarathy,[3] Hesham El Gamal,[4] R. Kelly Garrett,[5] Duane T. Wegener.[1]

[1] Department of Psychology, Ohio State University, Columbus, Ohio, United States

[2] Department of Psychology, Vanderbilt University, Nashville, Tennessee, United States

[3] Department of Computer Science and Engineering, Ohio State University, Columbus, Ohio, United States

[4] Faculty of Engineering, University of Sydney, Sydney, Australia

[5] School of Communication, Ohio State University, Columbus, Ohio, United States

* Corresponding author

E-mail: goebel.83@osu.edu (JTG)

Supplementary Data 1: Additional Analyses

Belief Shifts

Shifts in beliefs suggested that participants did pay attention to the information included in the update. In Study 1, there was a significant main effect of belief direction, such that those initially told that riskiness is good for firefighting generally shifted their beliefs in the direction of believing riskiness is bad, whereas those initially told riskiness is bad for firefighting generally shifted their beliefs towards believing riskiness is good, $b = -11.07$, $se = 1.79$, $t(218) = -6.17$, $p < .001$, 95% CI [-14.600, -7.534], $r = .39$. This overall difference was present because of a significant interaction between consistency and belief direction, $b = 20.98$, $se = 1.79$, $t(218) = 11.70$, $p < .001$, 95% CI [17.445, 24.511], $r = .62$. Those initially told riskiness is bad shifted their beliefs towards believing riskiness is good when the update information was inconsistent with their beliefs but showed minimal belief shifts when it was consistent with their beliefs, $b = 19.15$, $se = 2.55$, $t(218) = 7.52$, $p < .001$, 95% CI [14.132, 24.169], $r = .45$. Those told riskiness is bad shifted their beliefs towards believing it is good when the update information was inconsistent with their beliefs but demonstrated minimal belief shifts when it was consistent with their beliefs, $b = -22.81$, $se = 2.52$, $t(218) = -9.04$, $p < .001$, 95% CI [-27.780, -17.831], $r = .52$.

In Study 2 there was also a main effect of direction such that those initially told that the country should be allowed to join shifted their beliefs more in the direction of believing it should not be allowed to join than those initially told the country should not be allowed to join, $b = -8.11$, $se = 1.08$, $t(293) = -7.53$, $p < .001$, 95% CI [-10.237, -5.993], $r = .43$. Again, this overall difference was present because of a significant

interaction between consistency and belief direction, *b* = 13.22, *se* = 1.08, *t*(293) = 12.26, *p* < .001, 95% CI [11.099, 15.344], *r* = .58. Those initially induced to think the country should be allowed to join shifted their beliefs in the direction of believing the country should not be allowed to join when they received information inconsistent with their beliefs but showed minimal belief shifts when the information was consistent with their beliefs, *b* = 13.80, *se* = 1.50, *t*(293) = 9.18, *p* < .001, 95% CI [10.84, 16.76], *r* = .47. Those initially induced to think the country should not be allowed to join shifted their beliefs in the direction of believing that the country should be allowed to join when they received information inconsistent with their beliefs but minimally shifted their beliefs when the information was consistent with their beliefs, *b* = -12.64, *se* = 1.55, *t*(293) = -8.18, *p* < .001, 95% CI [-15.684, -9.600], *r* = .43.

Subjective Novelty and Surprise Predicting Sharing

We also examined whether participants' subjective ratings of novelty and surprise predict their likelihood of sharing information included in the update. Participants rated each piece of update information; these scores were subsequently averaged to create composites for our analyses. Subjective novelty and surprise were included as predictors in ordinal logistic regression models with the amount of update information shared as the outcome variable. Overall, results are equivocal as to the role of subjective novelty. Subjective surprise, however, negatively predicted sharing across both studies.

The association between subjective surprise and sharing reached significance in Study 1, *b* = -.33, *se* = .10, *t*(222) = -3.41, 95% CI [-.521, -.142], *OR* = 0.72, whereas the association between subjective novelty and sharing did not, *b* = .11, *se* = .09, *t*(222)

= 1.30, 95% CI [-.059, .288], *OR* = 1.12. In Study 2, there was again a negative association between surprise and sharing, *b* = -.36, *se* = .07, *t*(297) = -4.95, 95% CI [-.498, -.217], *OR* = 0.70. Yet in contrast to the prior study, a positive relation between novelty and sharing did reach significance, *b* = .31, *se* = .09, *t*(291) = 3.42, 95% CI [.132, .487], *OR* = 1.35.

Interaction Models (Examining Interactions of Update Information Direction and Belief Rather than Coded Belief Consistency – as Presented in the Main Text)

Experiment Analysis Strategy

Instead of coding the consistency of update information with manipulated initial beliefs (as presented in the main text), an alternative way to analyze the data would be to keep the direction of the update information as a separate variable from initial belief and examine interactions of those two factors. [In such an analysis, the interaction of update information direction and initial belief is the same as the belief consistency term in the main text analyses.] Most of the following analyses used multiple regression, with exceptions noted below. Predictors in these analyses were always the same: outcomes were predicted by the direction of the information provided in the update (hereafter "update direction"; riskiness is good/bad in Experiment 1 and the country should/should not be allowed to join the EU in Experiment 2), the novelty of the information, and the initial belief condition (riskiness is good/bad in Experiment 1 and the country should/should not be allowed to join the EU in Experiment 2), in addition to all two- and three-way interactions between these variables.

Manipulation Check

Our manipulations were effective in instilling the desired initial beliefs. In Experiment 1, those in the riskiness-is-good-for-firefighting condition indicated that they thought riskiness was significantly better for firefighting than those in the riskiness-is-bad-for-firefighting condition, $b = 23.28$, $se = 1.55$, $t(218) = 15.03$, $p < .001$, 95% CI [20.228, 26.334], $r = .71$. In Experiment 2, those induced to believe that the country should be allowed to join the EU indicated that they thought the country should be allowed to join significantly more than those induced to believe it should not be allowed to join, $b = 18.96$, $se = 1.23$, $t(293) = 15.45$, $p < .001$, 95% CI [16.55, 21.38], $r = .67$.

Outcome 1: Surprise

In Experiment 1, there was a significant interaction between update direction and initial belief condition, $b = -.61$, $se = .08$, $t(218) = -7.32$, $p < .001$, 95% CI [-.773, -.445], $r = .44$. Update information suggesting that riskiness is bad for firefighting was perceived as more surprising to people who initially believed that riskiness is good for firefighting ($M = 3.65$, $SD = 1.14$) than by people who initially believed that riskiness is bad for firefighting ($M = 2.49$, $SD = 1.17$), $b = .60$, $se = .12$, $t(218) = 4.91$, $p < .001$, 95% CI [.356, .834], $r = .32$. In contrast, update information suggesting that riskiness is good for firefighting was perceived as less surprising to people who initially believed that riskiness is good for firefighting ($M = 2.24$, $SD = 1.26$) than by people who initially believed that riskiness is bad for firefighting ($M = 3.50$, $SD = 1.33$), $b = -.62$, $se = .11$, $t(218) = -5.47$, $p < .001$, 95% CI [-.848, -.399], $r = .35$. A similar interaction emerged in Experiment 2, $b = -.69$, $se = .10$, $t(293) = -7.04$, $p < .001$, 95% CI [-.881, -.496], $r = .38$. Update information suggesting that the country should not join was perceived as more surprising by people who initially believed that the country should join ($M = 4.27$, $SD =$

1.77) than by people who initially believed that the country should not join (*M* = 2.86, *SD* = 1.59), *b* = .70, *se* = .13, *t*(293) = 5.30, *p* < .001, 95% CI [.442, .963], *r* = .30. In contrast, update information suggesting that the country should join was perceived as less surprising by people who initially believed that the country should join (*M* = 2.59, *SD* = 1.61) than by people who initially believed that the country should not join (*M* =3.98, *SD* = 1.75), *b* = -.68, *se* = .14, *t*(293) = -4.69, *p* < .001, 95% CI [-.958, -.391], *r* = .26. In other words, information consistent with the participant's initial beliefs elicited less surprise than belief-inconsistent information.

The novelty manipulation did not have parallel effects on surprise. The novelty manipulation did not impact surprise in Experiment 1, *b* = -.08, *se* = .08, *t*(218) = -0.98, *p* = .33, 95% CI [-.246, .082], *r* = .07, or in Experiment 2, *b* = .05, *se* = .10, *t*(293) = 0.49, *p* = .63, 95% CI [-.145, .240], *r* = .03. Of note, participants' ratings of novelty and their ratings of surprise were not positively correlated in either study as would be expected from semantic-free information theory. To the contrary, a small negative correlation was observed in both Experiment 1, *r*(224) = -.24, *p* < .001, and Experiment 2, *r*(299) = -.18, *p* = .002.

Outcome 2: Perceived Value and Sharing Intentions

In Experiment 2, there was a significant interaction between update direction and initial belief condition, *b* = .21, *se* = 07, *t*(293) = 2.99, *p* = .003, 95% CI [.073, .356], *r* = .17, such that people who initially believed that the country should join deemed update information suggesting that the country should join more valuable, *b* = .39, *se* = .10, *t*(293) = 3.69, *p* < .001, 95% CI [.182, .599], *r* = .21, but not update information suggesting the country should not join, *b* = -.04, *se* = .10, *t*(293) = -0.40, *p* = .69, 95% CI

[-.231, .152], *r* = .02. This seeming preference for belief-consistent information parallels the pattern of actual sharing decisions.

Since our information sharing outcome variable is a count of the amount of update information that was shared, these analyses were conducted using ordinal logistic regression. In Experiment 1, participants were more likely to share information suggesting that riskiness is good for firefighting than information suggesting that riskiness is bad, *b* = .33, *se* = .14, *t*(217) = 2.49, *OR* = 1.40, 95% CI [.073, .603]. More importantly, there was also a significant interaction between update direction and initial belief condition, *b* = .44, *se* = .14, *t*(217) = 3.25, *OR* = 1.55, 95% CI [.177, .709]. For both directions of update information, the amount of sharing was higher for belief-consistent than for belief-inconsistent information, though this tendency only reached significance for the update information suggesting that riskiness is bad for firefighting. That is, people who initially believed that riskiness is good for firefighting shared update information suggesting that riskiness is bad for firefighting significantly less than people who initially believed that riskiness is bad for firefighting, *b* = -.70, *se* = .19, 95% CI [-1.074, -.316]. In contrast, people who initially believed that riskiness is good for firefighting shared more update information suggesting that riskiness is good for firefighting, though this tendency did not reach significance, *b* = .19, *se* = .19, 95% CI [-.186, .556].

In Experiment 2, participants were more likely to share information suggesting that the country should join the EU compared to information saying it should not, *b* = .32, *se* = .13, *t*(292) = 2.58, *OR* = 1.38, 95% CI [.081, .575]. Critically, the interaction between update direction and initial belief condition emerged once more, *b* = .36, *se* =

.13, $t(292) = 2.87$, $OR = 1.44$, 95% CI [.115, .610]. Again, for both directions of update information, the amount of sharing was higher for belief-consistent than for belief-inconsistent information, though this tendency only reached significance for the update information that was less shared overall. People who initially believed that the country should join shared update information suggesting that the country should not join significantly less than people who initial believed that the country should not join, $b = -.39$, $se = .16$, 95% CI [-.699, -.080]. In contrast, people who initially believed that the country should join shared update information suggesting that the country should join more than people who initial believed that the country should not join, but this tendency did not reach significance, $b = .33$, $se = .20$, 95% CI [-.051, .717]. There was also an unanticipated interaction between update direction and novelty, $b = -.26$, $se = .13$, $t(292) = -2.04$, $OR = .77$, 95% CI [-.507, -.013], such that novel information was shared less often when update information indicated that the country should join, $b = -.41$, $se = .20$, 95% CI [-.790, -.022], but not when update information suggested that the country should not join, $b = .11$, $se = .16$, 95% CI [-.201, .416]. The level of novelty did not further moderate the key update direction by initial belief interactions.

Supplementary Figure 1. *Effect of update direction on the amount of update information shared moderated by initial belief direction.*

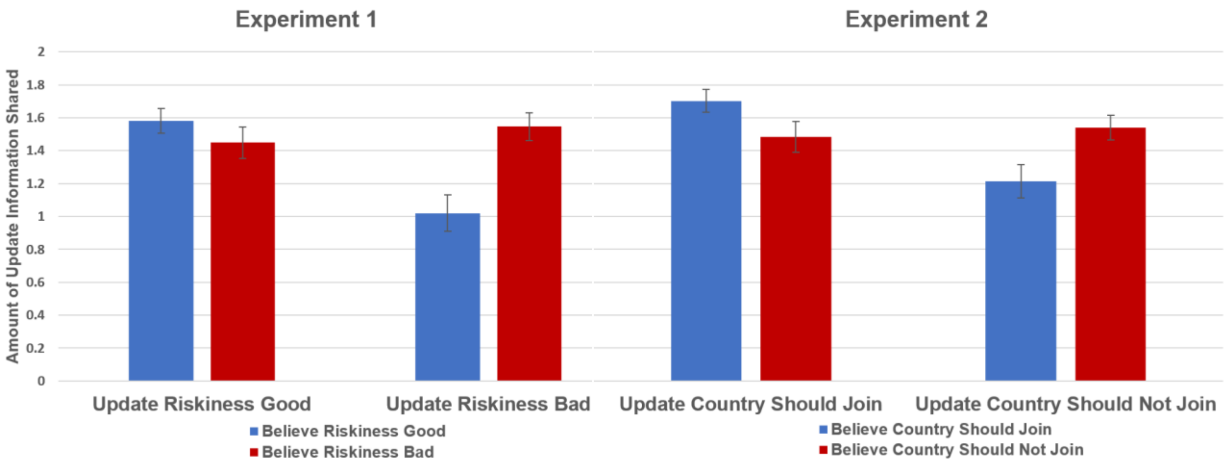

Note. Each bar indicates the mean amount of update information shared by participants. Error bars represent standard error.

Outcome 3: Perceived Novelty

We next tested whether subjective novelty ratings for the update information differed across conditions. The novelty manipulation had no significant effect on perceived novelty in Experiment 1, *b* = .08, *se* = .10, *t*(218) = 0.76, *p* = .45, 95% CI [-.126, .283], *r* = .05, or in Experiment 2, *b* = -.06, *se* = .08, *t*(293) = -0.75, *p* = .45, 95% CI [-.216, .097], *r* = .04, suggesting that participants did not consistently identify information they had not seen before as being more novel than information they had seen before. The interaction between update direction and initial belief condition did not reach significance in Experiment 1, *b* = .13, *se* = .10, *t*(218) = 1.23, *p* = .22, 95% CI [-.077, .333], *r* = .08, or in Experiment 2, *b* = .14, *se* = .08, *t*(293) = 1.70, *p* = .09, 95% CI [-.022, .292], *r* = .10, suggesting that our consistency manipulation had a negligible impact on perceived novelty.

Note: Across the reported analyses, tests of model assumptions were performed. For cases where assumptions were violated, applying appropriate corrections does not change the significance of results. All statistical tests were two-tailed.

Supplementary Data 2: Sample Tweets and Ideological Coding

In addition to the ideological slant of each Tweet, we also coded whether the focal event described by the tweet was liberal- or conservative-aligned (these tended to covary, though there were some instances in which a tweet about a liberal-aligned event was presented in a conservative-aligned way and vice versa). For instance, a tweet stating that “Biden celebrates 'Inflation Reduction Act' as food, rent prices climb” describes a liberal-aligned event, but with a conservative-aligned message slant. Both reported consistency analyses centered on the alignment of the tweet itself (rather than the event). The following list contains example Tweets with their associated event and slant coding.

1. Word of flight to Cancun from frozen Texas lands Senator Ted Cruz in hot water (Reuters) (Pro-liberal event, Pro-liberal slant)
2. New York Governor Andrew Cuomo accused of sexual harassment by former adviser (Newsweek) (Pro-conservative event, Pro-conservative slant)
3. President Joe Biden blamed Russian President Vladmir Putin for the country's record rises in inflation. (Newsweek) (Pro-conservative event, Pro-liberal slant)
4. BREAKING: U.S. Supreme Court overturns the landmark Roe v. Wade case, ending nearly 50 years of constitutional protections for abortion. Bans are expected in roughly half the states. (AP) (Pro-conservative event, Pro-conservative slant)
5. President Joe Biden, in an address after the U.S. Supreme Court overturned Roe v. Wade, says "the health and life of women in this nation are now at risk." (AP) (Pro-conservative event, Pro-liberal slant)

6. Biden celebrates 'Inflation Reduction Act' as food, rent prices climb (Reuters) (Pro-liberal event, Pro-conservative slant)
7. BREAKING: A judge temporarily blocked Ohio’s ban on virtually all abortions Wednesday, again pausing a law that took effect after federal abortion protections were overturned by the U.S. Supreme Court in June. (AP) (Pro-liberal event, Pro-liberal slant)
8. Biden forgives millions of student loans; critics fear inflation (Reuters) (Pro-liberal event, Pro-conservative slant)
9. President Joe Biden said the U.S. government will forgive $10,000 in student loans for millions of debt-saddled former college students, keeping a pledge he made in the 2020 campaign for the White House (Reuters) (Pro-liberal event, Pro-liberal slant)
